# How the Anaerobic Enteropathogen *Clostridioides difficile* Tolerates Low O_2_ Tensions

**DOI:** 10.1128/mBio.01559-20

**Published:** 2020-09-08

**Authors:** Nicolas Kint, Carolina Alves Feliciano, Maria C. Martins, Claire Morvan, Susana F. Fernandes, Filipe Folgosa, Bruno Dupuy, Miguel Texeira, Isabelle Martin-Verstraete

**Affiliations:** a Laboratoire Pathogenèses des Bactéries Anaérobies, Institut Pasteur, UMR CNRS 2001, Université de Paris, Paris, France; b Instituto de Tecnologia Química e Biológica António Xavier, Universidade Nova de Lisboa, Oeiras, Portugal; c Institut Universitaire de France; University of Delaware

**Keywords:** oxygen reductase, peroxide reductase, oxygen tolerance, anaerobes, stress response, sigmab

## Abstract

Clostridioides difficile is a major cause of diarrhea associated with antibiotherapy. After germination of C. difficile spores in the small intestine, vegetative cells are exposed to low oxygen (O_2_) tensions. While considered strictly anaerobic, C. difficile is able to grow in nonstrict anaerobic conditions (1 to 3% O_2_) and tolerates brief air exposure indicating that this bacterium harbors an arsenal of proteins involved in O_2_ detoxification and/or protection. Tolerance of C. difficile to low O_2_ tensions requires the presence of the alternative sigma factor, σ^B^, involved in the general stress response. Among the genes positively controlled by σ^B^, four encode proteins likely involved in O_2_ detoxification: two flavodiiron proteins (FdpA and FdpF) and two reverse rubrerythrins (revRbr1 and revRbr2). As previously observed for FdpF, we showed that both purified revRbr1 and revRbr2 harbor NADH-linked O_2_- and H_2_O_2_-reductase activities *in vitro*, while purified FdpA mainly acts as an O_2_-reductase. The growth of a *fdpA* mutant is affected at 0.4% O_2_, while inactivation of both revRbrs leads to a growth defect above 0.1% O_2_. O_2_-reductase activities of these different proteins are additive since the quadruple mutant displays a stronger phenotype when exposed to low O_2_ tensions compared to the triple mutants. Our results demonstrate a key role for revRbrs, FdpF, and FdpA proteins in the ability of C. difficile to grow in the presence of physiological O_2_ tensions such as those encountered in the colon.

## Abstract (precis)

Although the gastrointestinal tract is regarded as mainly anoxic, low O_2_ tension is present in the gut and tends to increase following antibiotic-induced disruption of the host microbiota. Two decreasing O_2_ gradients are observed, a longitudinal one from the small to the large intestine and a second one from the intestinal epithelium toward the colon lumen. Thus, O_2_ concentration fluctuations within the gastrointestinal tract are a challenge for anaerobic bacteria such as C. difficile. This enteropathogen has developed efficient strategies to detoxify O_2_. In this work, we identified reverse rubrerythrins and flavodiiron proteins as key actors for O_2_ tolerance in C. difficile. These enzymes are responsible for the reduction of O_2_ protecting C. difficile vegetative cells from associated damages. Original and complex detoxification pathways involving O_2_-reductases are crucial in the ability of C. difficile to tolerate O_2_ and survive to O_2_ concentrations encountered in the gastrointestinal tract.

## INTRODUCTION

Clostridioides difficile (formerly Clostridium difficile) is a spore-forming and anaerobic Gram-positive bacterium. C. difficile’s aeroresistant and metabolically dormant spores are ubiquitous in the environment and are commonly found on hospital surfaces and in the mammalian gastrointestinal tract (1). Nowadays, this pathogen is considered the major cause of antibiotic-associated diarrhea and pseudomembranous colitis (2). C. difficile infection (CDI) is transmitted via the oral-fecal route (3) through ingestion of spores, the form for the transmission, resistance, and persistence of this bacterium (4). Colonization of the gastrointestinal tract usually occurs following antibiotic-induced dysbiosis of the host microbiota, which leads to substantial changes in the metabolic pool, particularly in bile acids (5). These metabolic modifications enable germination of the spores in the small intestine and thereafter colonization of the intestinal tract by the vegetative cells (6). Then, C. difficile cells produce virulence factors, including two toxins, TcdA and TcdB, that cause the pathology associated with CDI. These toxins are responsible for alteration of the actin cytoskeleton of intestinal epithelial cells, which induces intestinal cell lysis and triggers an important inflammation (7, 8). The inflammation process results in the production by the host immune system of reactive oxygen species (ROS), nitric oxide (NO), and reactive nitrogen species (RNS) at bactericidal concentrations (9, 10).

Apart from inflammation-induced oxidative and nitrosative stresses, oxygen (O_2_) is also a major stress that C. difficile faces during gut colonization. Indeed, although a healthy gut, with a diverse microbiota, is regarded as mainly anoxic, a longitudinal decreasing O_2_ gradient is observed along the gastrointestinal tract (11). From 4% in the small intestine lumen, which is the location of spore germination, the O_2_ tension decreases to 0.1 to 0.4% in the large intestine lumen, where vegetative cells multiply (11). A second increasing O_2_ gradient from the colon lumen toward the intestinal epithelium also occurs (from 0.1 to 0.4% to 5%) (12, 13). In addition, antibiotic-induced disruption of the host microbiota leads to an increased O_2_ level within the gut (12, 14). Thus, O_2_ concentration fluctuations within the gastrointestinal tract present a challenge to anaerobic bacteria such as C. difficile. While strictly anaerobic, C. difficile is able to grow in nonstrict anoxic conditions (1 to 3% O_2_) (15), indicating that this bacterium encodes an arsenal of proteins involved in O_2_ detoxification and protection against oxidative stress. Recently, the deletion of the *iscS2* gene encoding a cysteine desulfurase likely involved in Fe-S cluster biogenesis was shown to cause a severe growth defect in the presence of 2% O_2_ (16). Another essential actor in the C. difficile ability to tolerate low O_2_ concentrations is the alternative sigma factor involved in the general stress response, σ^B^. Indeed, a *sigB* mutant is unable to grow in the presence of 0.1% O_2_, a tension lower than that physiologically found in the large intestine (17). Of note, both *sigB* and *iscS2* mutants present a colonization defect in axenic mice, and a delay of colonization is observed for the *iscS2* mutant in conventional mice (17). These results suggest that O_2_ tolerance might be an important mechanism during the C. difficile colonization process. However, little is known about the proteins involved in the ability of C. difficile to tolerate O_2_.

In other organisms, both flavodiiron proteins (FDPs) and rubrerythrins (Rbrs) play an important role in protecting the cells from O_2_, oxidative, or nitrosative stresses (18–21). FDPs are enzymes composed of a minimal core containing a diiron catalytic center in a metallo-β-lactamase-like domain and a flavin-mononucleotide (FMN)-containing flavodoxin domain, located at the N- and C-terminal ends, respectively, of the protein (22–24). FDPs reduce oxygen to water or NO to nitrous oxide. Although some of these enzymes show substrate specificity toward O_2_ or NO, others have a dual activity.

Canonical Rbrs are composed by two types of iron sites: an N-terminal four-helix bundle domain harboring a non-sulfur diiron center and a rubredoxin (Rd)-like [Fe(SCys)_4_] domain in the C-terminal part (25, 26). In reverse Rbrs (revRbrs), the position of these two domains are reversed. Rbrs act as peroxidases through NAD(P)H oxidation in partnership with another redox partner, and O_2_-reductase activity has also been reported for a revRbr from Clostridium acetobutylicum (20, 25, 27, 28). Both FDPs and Rbrs are broadly distributed among anaerobic and microaerophilic bacteria and archaea, but they are also present in aerobes and in eukaryotes. Most FDPs, as well as Rbrs and revRbrs, require electron transfer proteins as physiological partners to couple the oxidation of NAD(P)H to the reduction of their substrates, such as rubredoxins (Rds), NAD(P)H/FAD dependent oxidoreductases or the F_420_ coenzyme in methanogens (20, 29–32).

The genome of C. difficile harbors genes encoding two FDPs (CD1157 and CD1623, here named FdpA and FdpF, respectively, according to their domain composition [24]), two Rbrs (CD0825 and CD2845) and two revRbrs (CD1474 and CD1524, here named revRbr1 and revRbr2, respectively). While *CD2845* is expressed under the control of σ^G^ (33) and its product is present in the spore (34), the expression of *CD0825*, *revRbr1*, *revRbr2*, *fdpA*, and *fdpF* is positively controlled by σ^B^ (17). Among the six aforementioned proteins, only FdpF is functionally characterized (35). FdpF is a large class F FDP (24) that harbors, in addition to the minimal core universally present in FDPs, two other domains: a Rd domain of the short type, which distinguishes from canonical Rds by its smaller number of amino acids in between the two pairs of iron-binding cysteines (∼12 amino acids versus ∼30 amino acids) (36) and a NAD(P)H:Rd oxidoreductase-like domain (NROR) (see Fig. S1 in the supplemental material). This protein acts mainly as an NADH:O_2_ oxidoreductase reducing O_2_ to H_2_O but also exhibits a detectable H_2_O_2_ reductase activity (35). Due to the presence in the same polypeptide chain of the extra domains, FdpF receives electrons directly from NADH and transfers them to O_2_ without the participation of additional protein partners (35). In the present study, we demonstrate a key role for both FDPs and both revRbrs, whose genes are controlled by σ^B^, in the ability of C. difficile to tolerate physiological O_2_ tensions encountered within the gut.

10.1128/mBio.01559-20.1 FIG S1 FDPs and revRbrs amino acids sequences analysis. (A) Domain organization of the FDPs and revRbrs. For FdpA and FdpF, the diiron center domain (1 to 248 amino acids) is represented in orange, and the flavodoxin domain (249 to 397 amino acids) is represented in yellow. For FdpF, the rubredoxin domain is represented in red, and the NADH:Rd oxidoreductase domain (NROR) is represented in pale yellow. For revRbrs, the Rd domains (1 to 34 amino acids) are represented in red, and the diiron center containing domain (35 to 181 amino acids) is represented in orange. (B) Amino acid sequence alignment of revRbr1 and revRbr2. The two CysXXCys motifs from the [Fe(SCys)_4_] center of the Rd domain are highlighted in black. The alignment was performed with ClustalW (77). Download FIG S1, TIF file, 1.2 MB. Copyright © 2020 Kint et al. 2020 Kint et al. This content is distributed under the terms of the Creative Commons Attribution 4.0 International license.

## RESULTS

### Regulation of *revRbr1*, *revRbr2*, *fdpF*, and *fdpA* expression.

**FIG 1:**
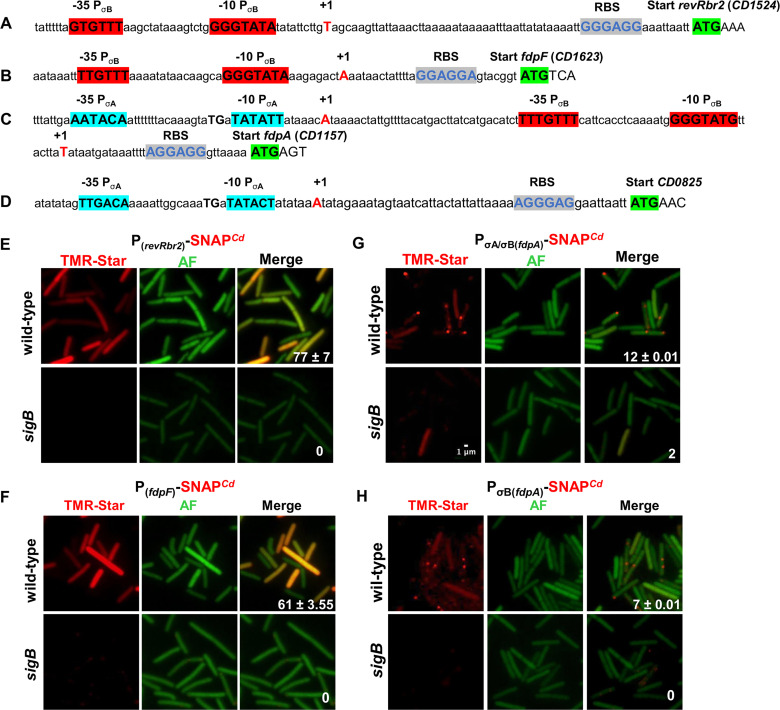
Transcriptional control of *revRbr2*, *fdpF*, and *fdpA*. The promoter regions of *revRbr2* (A), *fdpF* (B), *fdpA* (C), and *CD0825* (D) genes are shown. The mapped transcriptional start sites (+1) located at positions 1766842 (*revRbr2*), 1878917 (*fdpF*), 1356839 and 1356915 (*fdpA*), and 1000259 (*CD0825*) are represented in red uppercase. The −10 and −35 promoter elements corresponding to the consensus for promoters recognized by σ^A^ or σ^B^ are indicated in boldface and highlighted in blue and red, respectively (37). The possible RBS and start codon of *revRbr1*, *fdpF*, *fdpA*, and *CD0825* are highlighted in blue and green, respectively. Microscopy analysis of C. difficile cells carrying P*_revRbr2_*-SNAP*^Cd^* (E), P*_fdpF_*-SNAP*^Cd^* (F), P_σA/σB(_*_fdpA_*_)_-SNAP*^Cd^* (G), and P_σB(_*_fdpA_*_)_-SNAP*^Cd^* (H) transcriptional fusions in the 630Δ*erm* strain and in the *sigB* mutant was performed. The strains were grown 16 h in BHI. The merged images show the overlap between the TMR-Star (red) and the autofluorescence (AF; green) channels. About 200 cells were scored for each strain and the numbers represent the percentage of cells with signal. These data are representative of two independent experiments. Scale bar, 1 μm.

Genome-wide transcriptional start site (TSS) mapping allowed us to identify promoters upstream of *CD0825*, *revRbr1* (CD1474), *revRbr2* (CD1524), *fdpA* (CD1157), and *fdpF* (CD1623) (37). A σ^B^ consensus sequence [WGWTT-N_13-17_-(G/T)GGTAWA] was identified upstream of the TSSs mapped in the promoter region of *revRbr1*, *revRbr2*, *fdpF*, and *fdpA* genes (Fig. 1A to C) (17). In contrast, a canonical −10 box (TATACT) and a −35 box (TTGACA) of a σ^A^-dependent promoter were identified upstream of the *CD0825* mapped TSS (Fig. 1D), suggesting indirect control by σ^B^, in agreement with the weaker transcriptional control by σ^B^ observed for this gene compared to the others (17). Interestingly, upstream of the σ^B^-dependent promoter of the *fdpA* gene, a second TSS was mapped corresponding to a promoter with a canonical extended −10 box (TGNTATATT) of a σ^A^-dependent promoter and a poorly conserved −35 box, as often observed with a −10 extended box (Fig. 1C).

We next focused on the genes harboring a σ^B^ consensus sequence in their promoter region, and we wanted to determine whether their expression was strictly dependent on σ^B^. Using transcriptional fusions between the promoter regions of the *revRbr2* and *fdpF* genes and the gene encoding the fluorescent reporter SNAP*^Cd^* (38), we detected expression of the P*_revRbr2_*- and P*_fdpF_*-SNAP*^Cd^* fusions in the wild-type (WT) strain in 77 and 61% of the cells, respectively, after 16 h of growth. In contrast, the expression of both fusions was completely abolished in the *sigB* mutant as observed for the *revRbr1* gene (Fig. 1E and F) (39). To study the more complex expression of the *fdpA* gene from its two promoters, we first constructed a transcriptional fusion between SNAP*^Cd^* and the two promoters. Expression of the P_σA/σB(*fdpA*)_-SNAP*^Cd^* fusion was detected in 12% of the cells in the WT strain (Fig. 1G). Contrary to the P*_revRbr2_*- and P*_fdpF_*-SNAP*^Cd^* fusions, expression of the P_σA/σB(*fdpA*)_-SNAP*^Cd^* fusion in the *sigB* mutant was still detected in a small fraction of the cells in agreement with the existence of the σ^A^-dependent promoter (Fig. 1G). When we monitored the expression of a P_σB(_*_fdpA_*_)_-SNAP*^Cd^* fusion containing only the σ^B^-dependent promoter, we detected fluorescent cells in the WT strain but not in the *sigB* mutant, confirming that this promoter is recognized by σ^B^ (Fig. 1H). Thus, the *fdpA* expression is dependent on both σ^A^ and σ^B^, while the expression of *revRbr2* and *fdpF* is only σ^B^ dependent in C. difficile, as observed for *revRbr1* (39).

### FDPs and revRbrs of *C. difficile*.

FdpA (397 amino acids) is a class A FDP (see Fig. S1A in the supplemental material) harboring only the minimal core common to all FDPs (24). The sequence alignment of both FdpA and FdpF cores (diiron and FMN domains) against the structural alignment of all FDPs with available structures (see Fig. S2A) reveals that the sequence identity between the core domains of FdpA and FdpF is only 29%. Despite this relatively low identity percentage, the structural alignment shows that the most conserved regions of characterized FDPs are also present in both FdpA and FdpF. The predicted amino acids involved in the coordination of the diiron center in FdpA are histidines (His^80^, His^85^, His^146^, and His^224^), aspartates (Asp^84^ and Asp^165^), and one glutamate (Glu^82^) (see Fig. S2A) (35). The sequence alignment also reveals a significant conservation of other features, such as the putative chain of aromatic amino acids (22) formed by Tyr^191^, Tyr^192^, Tyr^239^, and Trp^242^.

10.1128/mBio.01559-20.2 FIG S2 Amino acid sequence alignment of the cores of FDPs and both rubrerythrins and reverse rubrerythrins. (A) The amino acid sequence alignment and the secondary structure prediction were based on structural superpositions of FDPs available structures using Chimera (78). Then, ClustalX (79) in Profile Alignment Mode was used to align the sequence of the C. difficile FdpA and FdpF core domains (residues 1 to 397). Amino acid residues that coordinate the catalytic iron atoms are marked with stars and highlighted with blue boxes. Strictly conserved amino acids represented as black boxes, whereas dark grey boxes represent the mostly conserved residues among the selected sequences. Below the sequences is represented the predicted secondary structure. The amino acids that are part of the conserved aromatic residues chain are marked with orange boxes. Besides the C. difficile FDPs, the sequences represented in the alignment are the following: *D. gigas*, Desulfovibrio gigas (PDB 1E5D); T. maritima, Thermotoga maritima (PDB 1VME); *M. thermoacetica*, Moorella thermoacetica (PDB 1YCF); *Mt. marburgensis*, Methanothermobacter marburgensis (PDB 2OHH); *G. intestinalis*, Giardia intestinalis (PDB 2Q9U), and E. coli, Escherichia coli (PDB 4D02). (B) The amino acid sequence alignment was based on structural superpositions of Rbrs available structures using Chimera, excluding those with swapped domains Then, ClustalX (79) in Profile Alignment Mode was used to align the sequence of the C. difficile revRbr1 and revRbr2 four-helix bundle domains (residues 35 to 147). Amino acid residues that coordinate the catalytic iron atoms are highlighted with blue boxes. Strictly conserved amino acids represented as black boxes, whereas dark grey boxes represent the mostly conserved residues among the selected sequences. Besides C. difficile revRbrs, the sequences represented in the alignment are the following: rubrerythrin *D. vulgaris*, Desulfovibrio vulgaris Hildenborough rubrerythrin (PDB 1LKO); Nigerythrin *D. vulgaris*, Desulfovibrio vulgaris Hildenborough nigerythrin (PDB 1YV1), and Symerythrin *Cy. paradoxa*, *Cyanophora paradoxa* (PDB 3QHC). Download FIG S2, TIF file, 2.4 MB. Copyright © 2020 Kint et al. 2020 Kint et al. This content is distributed under the terms of the Creative Commons Attribution 4.0 International license.

RevRbr1 and revRbr2 share 96% amino acid identity among themselves (Fig. S1B). However, only ca. 25% identity is observed with other rubrerythrins, when a sequence alignment of the four-helix bundle domain of these two revRbrs is performed against a structural alignment of the four-helix bundle domain of three canonical rubrerythrins with known structures (see Fig. S2B). The major predicted difference occurs in the loop connecting the two pairs of helices, which is shorter in revRbrs. Contrary to what was observed for the C. difficile FDPs, there is no significative conservation of large protein regions apart from the diiron center ligands (see Fig. S2B), which are predicted to be glutamates (Glu^67^ and Glu^130^) and histidines (His^70^ and His^133^, revRbr1 numbering) (26). The Rd domain contains two CysXXCys motifs (Cys^6^/Cys^9^ and Cys^22^/Cys^25^) separated by 12 residues (see Fig. S1B), which is typical of short-spaced Rd domains found in most Rbrs, as well as in the Rd domains of several classes of FDPs, including FdpF (24, 35, 36).

### Proteins characterization and redox properties.

To characterize their properties and functions, the FdpA, revRbr1, and revRbr2 proteins were overexpressed in Escherichia coli and purified through several chromatographic steps in a process analogous to those previously used for similar proteins (32, 40–46). The molecular masses determined by SDS-PAGE were ∼45 kDa for FdpA and ∼22 kDa for the two revRbrs, in agreement with those calculated from their respective amino acid sequences, 44.8 and 20 kDa, respectively (see Fig. S3A). Size exclusion chromatography of the purified proteins revealed that FdpA is a homodimer in solution, with a molecular mass of ∼82 kDa, while both revRbrs are isolated as tetramers with molecular masses of ∼90 kDa (see Fig. S3B). The presence of homodimers and homotetramers in solution is typical of other FDPs and Rbrs (23, 47, 48). For FDPs, the homodimer organization is essential for efficient electron transfer between the electron accepting site in one monomer, the FMN, and the diiron center of the other monomer.

10.1128/mBio.01559-20.3 FIG S3 Study of revRbr1, revRbr2, and FdpA purified from E. coli. (A) SDS-PAGE gel of purified revRbr1, revRbr2, and FdpA from E. coli. Low-range molecular mass markers are indicated. (B) Size exclusion chromatogram of FdpA and revRbrs. The lines represent the chromatograms obtained from the size exclusion chromatography for FdpA (point-dash line), revRbr1 (dash line), and revRbr2 (solid line), monitored at 280 nm. We used a Superdex S200 10/300 GL column (GE Healthcare) previously equilibrated with buffer A containing 150 mM NaCl. The arrow in panel A points to the dextran blue elution band, while the arrow in panel B points to the protein’s elution bands. * indicates a small amount of free flavin. Download FIG S3, TIF file, 0.2 MB. Copyright © 2020 Kint et al. 2020 Kint et al. This content is distributed under the terms of the Creative Commons Attribution 4.0 International license.

For FdpA, quantification of iron and flavin yielded iron/FMN/protein monomer ratios of 1/0.3/1. These values are lower than expected (i.e., 2/1/1), indicating an incomplete incorporation of both iron and FMN. Even after incubation with FMN following purification, no significant improvement was observed. For revRbr1 and revRbr2, the iron/protein monomer ratios were 2.1/1 and 2.9/1, respectively, instead of the expected 3/1 ratio. Thus, while revRbr2 is almost fully loaded with iron, revRbr1 is only partially loaded.

**FIG 2:**
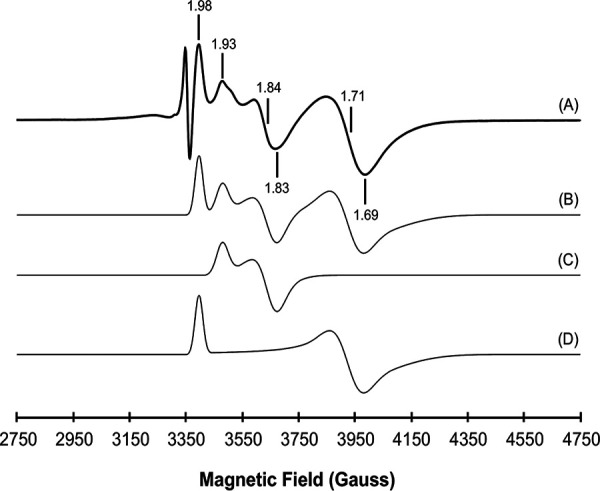
Electron paramagnetic resonance spectra of FdpA. Spectrum A corresponds to FdpA after anaerobic incubation with one equivalent of menadiol. The two species observed in the experimental spectrum were theoretically simulated (spectrum C with g = 1.83, 1.84, and 1.93; spectrum D with g = 1.69, 1.71, and 1.98), and spectrum B corresponds to their sum at a 1:0.9 ratio. Temperature of 7 K, microwave frequency of 9.41 GHz, modulation amplitude of 1.0 mT, and microwave frequency of 2 mW.

By reversed-phase high-pressure liquid chromatography, FMN was identified as the FdpA flavin cofactor, as observed so far for all FDPs (22, 24). FdpA exhibited a UV-visible absorbance spectrum typical of a flavin-containing protein, with maxima at 380 and 450 nm (see Fig. S4A). The UV-visible spectra of revRbr1 and revRbr2 were characteristic of proteins containing a Rd center, with maxima at 380, 490, and 580 nm (see Fig. S4B and C). For these three proteins, the spectral contribution of the diiron sites was not detected due to their low molar absorptivities (49). Nevertheless, the diiron site of FdpA was detected by electron paramagnetic resonance (EPR) upon partial reduction with menadiol. As already observed for the E. coli FDP (50), the EPR spectrum revealed a mixture of species, with g values of 1.69, 1.71, 1.98, 1.83, 1.84, and 1.93 (Fig. 2), typical of S = 1/2 anti-ferro-magnetically coupled diiron sites in the mixed valence form [Fe(III)-Fe(II)]. For the revRbrs, we could only observe the typical resonances at g ≈ 4.3 and a minor resonance at g ≈ 9.3, attributable, respectively, to the middle (|±3/2>) and lower (|±1/2>) doublets of a high-spin (S = 5/2) ferric center, with a rhombicity (E/D) close to 0.33, characteristic of the ferric [Fe(SCys)_4_] sites (51). Overall, the spectroscopic properties of FdpA and revRbrs are identical to those of analogs (42–44).

10.1128/mBio.01559-20.4 FIG S4 UV-visible spectra of FdpA, revRbr1, and revRbr2. The full lines represent the spectra of the as isolated proteins FdpA (A), revRbr1 (B), and revRbr2 (C). The insets correspond to the spectra of the reduced proteins after incubation using E. coli Rd-D (2.5 μM for the revRbrs or 5 μM for FdpA assays) and NROR (2.5 μM for the revRbrs or 5 μM for FdpA assays) upon addition of 200 μM of NADH. For all spectra, the protein concentration was 30 μM in 50 mM Tris-HCl (pH 7.5) containing 18% glycerol. Download FIG S4, TIF file, 0.6 MB. Copyright © 2020 Kint et al. 2020 Kint et al. This content is distributed under the terms of the Creative Commons Attribution 4.0 International license.

The reduction potential of the Rd center for both revRbrs was obtained by redox titrations monitored by visible spectroscopy in an anaerobic chamber at pH 7.5, following the decrease in absorbance of the ferric Rd site (see Fig. S5A and B). We obtained values of +10 mV ± 5 mV and of −10 mV ± 5 mV for revRbr1 and revRbr2, respectively, which are similar to those of previously isolated Rds (generally in the range of −50 to +50 mV [reviewed in reference 51]) but significantly lower than those thus far reported for the Rd centers of the few studied canonical rubrerythrins: +213 and +281 mV for the Desulfovibrio vulgaris nigerythrin and rubrerythrin, respectively, and +185 mV for the Campylobacter jejuni desulforubrerythrin (43, 47, 52). Interestingly, for the enzyme from C. jejuni, the desulforedoxin-like center has a high positive potential (+240 mV) (47), compared to other known desulforedoxin-like centers (∼0 mV). This reinforces the idea that there is a very large variability in the reduction potentials of Rd and Rd-like centers that remain to be assigned to specific features of the polypeptide chains close to the metal center, in spite of its identical structure. The flavin center of FdpA was titrated similarly, and the reduction potentials obtained were −60 ± 15 and −175 ± 15 mV for the FMN_ox_/FMN_semiquinone_ and FMN_semiquinone_/FMN_red_ redox transitions (see Fig. S5C), values within the range of those available for a few other FDPs (24, 53).

10.1128/mBio.01559-20.5 FIG S5 Anaerobic redox titration curves of revRbr1, revRbr2, and FdpA. Panels correspond to the normalized intensities measured at 490 nm to follow the Rd domain of revRbr1 (A) and revRbr2 (B) and 450 nm to follow the reduction of the FMN cofactor of FdpA (C). In all titrations, protein concentration was 30 μM, and the experiments were performed in 50 mM Tris-HCl (pH 7.5) containing 18% glycerol. The solid lines correspond to fits to the experimental data using the Nernst equations adjusted as described in Materials and Methods, with the following reduction potentials: revRbr1, +10 mV; revRbr2, −10 mV; and FdpA, −60 and −175 mV. Download FIG S5, TIF file, 0.4 MB. Copyright © 2020 Kint et al. 2020 Kint et al. This content is distributed under the terms of the Creative Commons Attribution 4.0 International license.

### revRbrs and FDP H_2_O_2_- and O_2_-reductase activities.

**FIG 3:**
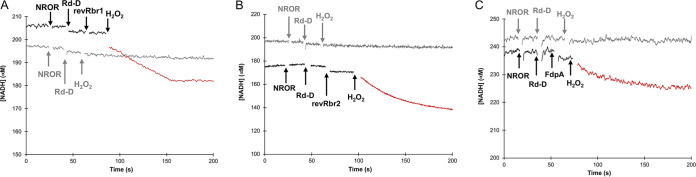
H_2_O_2_-reductase activity of revRbr1, revRbr2, and FdpA. The activities were determined anaerobically (at least five assays for each enzyme) by measuring NADH consumption monitored at 340 nm. The data presented are representative of the results obtained. The protein concentrations in each assay were 2.5 μM for NROR, 2.5 μM for the Rd-D, and 0.1 μM for revRbr1 (A) and 0.03 μM for revRbr2 (B). (C) In the assays where FdpA was tested, the protein concentrations were 5 μM for the NROR and the Rd-D and 1 μM for FdpA. Experiments were performed in the presence of 200 μM NADH and different H_2_O_2_ concentrations: 30 μM and 90 μM H_2_O_2_ were used for revRbr1 and revRbr2, respectively, and 50 μM H_2_O_2_ for FdpA. Arrows indicate the time points of the successive additions of NROR, Rd-D, revRbrs or FdpA, and H_2_O_2_. Black and gray curves represent the reaction in the presence or absence of revRbrs or FdpA, respectively. Red curves correspond to the part with all the products added. The H_2_O_2_-reductase activity rates (s^−1^) resulted from the subtraction of the experimental slope (μM/s) before and after the addition (represented in a different color) of the substrate, H_2_O_2_, divided by the protein concentration (μM).

Having established the presence of the FMN and iron cofactors and determined the redox properties of FdpA, revRbr1, and revRbr2, we addressed the catalytic activities of these proteins. The O_2_- and H_2_O_2_-reductase activities of the three enzymes were determined using NADH as the primary electron donor. As expected, FdpA, revRbr1, and revRbr2 alone did not show any NADH oxidase activity (Fig. 3, before the addition of substrate). Since the C. difficile physiological electron donors of these proteins are still unknown and its genome does not encode any Rd that could act as a redox partner, as happens in a few organisms, we used a heterologous system to perform the assays. We found that the truncated Rd domain (Rd-D) of E. coli FDP (flavoRd) and its reductase, homologous to NADH:Rd oxidoreductases (NROR), were able to reduce FdpA and the two revRbrs in the presence of NADH (see Fig. S4, insets).

To test the H_2_O_2_-reductase activity, we spectrophotometrically measured the NADH consumption upon addition of H_2_O_2_ to a premix containing NADH, NROR, Rd-D, and either FdpA, revRbr1, or revRbr2. A clear H_2_O_2_-reductase activity was observed for revRbr1 and revRbr2 with rates of 2.1 ± 0.1 s^−1^ and 7.7 ± 0.7 s^−1^, respectively (Fig. 3A and B; Table 1). The reduced activity detected for revRbr1 might be related to its lower content of iron compared to revRbr2. In contrast, we detected only a negligible H_2_O_2_-reductase activity with a rate of 0.27 ± 0.03 s^−1^ for FdpA (Fig. 3C and Table 1). It should be mentioned that thus far such activity was reported only for the FdpF from C. difficile (35).

**TABLE 1:** NADH linked H_2_O_2_- and O_2_-reductase activities of FDPs and revRbrs^*a*^

Gene	Name	Avg activity ± SD (s^−1^)		
		NADH:H_2_O_2_-reductase	NADH:O_2_-reductase	NADH:NO-reductase
*CD1157*	FdpA	0.27 ± 0.03	1.4 ± 0.3	0.16 ± 0.07
*CD1474*	revRbr1	2.1 ± 0.1	1.2 ± 0.1	
*CD1524*	revRbr2	7.7 ± 0.7	2.0 ± 0.4	

a NADH-linked H_2_O_2_ and NADH-linked O_2_ reductase activities were determined in the presence of NADH, Rd-D and NROR for FdpA, revRbr1, and revRbr2. The concentration of NADH used was 200 μM for the H_2_O_2_-reductase activity and 5 mM for the O_2_-reductase and the NO-reductase activities. For H_2_O_2_-reductase activity, the rates were calculated subtracting the experimental slope (μM/s) before and after the addition of H_2_O_2_, divided by the protein concentration (μM). For O_2_-reductase activity, the rates were calculated subtracting the experimental slope (μM/s) before and after the addition of each enzyme, divided by its concentration (μM). For NO-reductase activity, the calculated rates were calculated subtracting the experimental slope (μM/s) before and after the addition of FdpA, divided by its concentration (μM). The data are averages of the calculated rates of five experiments (H_2_O_2_-reductase), six experiments (O_2_-reductase), and six experiments (NO-reductase) with the standard deviations.

**FIG 4:**
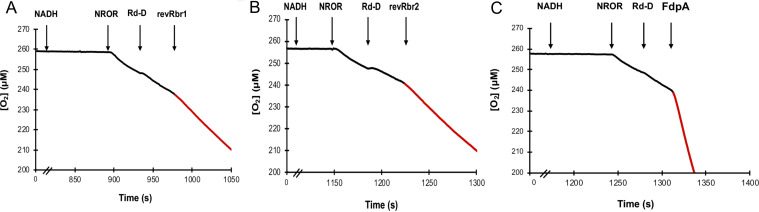
O_2_-reductase activity of revRbr1, revRbr2 and FdpA. At least six assays for each enzyme were performed using a Clark-type electrode selective for O_2_. The data presented are representative of the results obtained. Each assay contained 5 mM NADH as electron donor, and protein concentrations were 2.5 μM for NROR and Rd-D, 0.1 μM for both revRbr1 (A) and revRbr2 (B), and 1 μM for FdpA (C). Arrows indicate the time points of successive additions of NADH, NROR, Rd-D and revRbr1, revRbr2 or FdpA. The O_2_-reductase activity rates (s^−1^) measured in air-saturated buffer (ca. 260 μM O_2_) resulted from the subtraction of the experimental slope (μM/s) before and after the addition (represented in red) of each enzyme, divided by its concentration (μM of enzyme).

The O_2_-reductase activity was measured by following O_2_ consumption using an O_2_ Clark-type selective electrode. FdpA, revRbr1, or revRbr2 were added to an air-saturated buffer (∼260 μM O_2_) containing both NROR and Rd-D from E. coli and an excess of NADH (5 mM). We observed an O_2_-reductase activity for FdpA, revRbr1, and revRbr2 with rates of 1.4 ± 0.3 s^−1^, 1.2 ± 0.1 s^−1^, and 2.0 ± 0.4 s^−1^, respectively (Fig. 4 and Table 1). These activities may be underestimated since the physiological partners in C. difficile were not used in the assays as they remain to be identified. However, we can conclude from these results that FdpA acts as an NADH-linked O_2_-reductase and that the two revRbrs act both as NADH-linked H_2_O_2_-reductase and NADH-linked O_2_-reductase *in vitro*. Furthermore, FdpA exhibits a negligible NO reductase activity, 10-fold lower than for O_2_ reduction (see Fig. S6A). Thus, like FdpF, FdpA is selective for O_2_.

10.1128/mBio.01559-20.6 FIG S6 NO-reductase activity of FdpA and H_2_O_2_ sensitivity of mutants inactivated for revRbrs- and FDPs-encoding genes. (A) At least 6 assays were performed using a Clark-type electrode selective for NO. The data presented are representative of the results obtained. The NO reductase activity was evaluated at 25°C in degassed buffer, in the presence of an O_2_ scavenging system (10 mM glucose, 375 nM glucose oxidase, and 750 nM catalase), NROR (2.5 μM), and Rd-D (2.5 μM). Sequential additions of NO (up to 12 μM) were followed by the addition of 5 mM NADH, and the reaction was initiated by the addition of FdpA (0.1 μM). Arrows indicate the time points of successive additions of NO, NADH, NROR, Rd-D, and FdpA. (B) The histograms represent the growth inhibition diameter of the 630Δ*erm* (black), the Δ*revRbr1/2* double mutant (white), the Δ*revRbr1/2-ΔfdpF* triple mutant (light grey), and the Δ*revRbr1/2-ΔfdpF-fdpA*::*erm* quadruple mutant (dark grey). These results are the means with the standard deviations of seven experiments. Download FIG S6, TIF file, 0.2 MB. Copyright © 2020 Kint et al. 2020 Kint et al. This content is distributed under the terms of the Creative Commons Attribution 4.0 International license.

### Involvement of revRbrs and FDPs in H_2_O_2_ stress management.

To study the physiological role of the two revRbrs and the two FDPs in C. difficile, we deleted the genes encoding revRbr1, revRbr2, and FdpF using the allelic chromosomic exchange (ACE). We generated Δ*revRbr1*, Δ*revRbr2*, and Δ*fdpF* single mutants and a Δ*revRbr1/2* double mutant, as well as a Δ*revRbr1/2-*Δ*fdpF* triple mutant. We also inactivated *fdpA* by insertion of an intron into this gene using the clostron system to generate a *fdpA* single mutant, a *fdpA*-Δ*fdpF* double mutant, a Δ*revRbr1/2*-*fdpA* triple mutant, and a Δ*revRbr1/2*-Δ*fdpF*-*fdpA* quadruple mutant. Since we showed that revRbr1 and revRbr2, as well as FdpF (35), have significant *in vitro* H_2_O_2_-reductase activities (Table 1), we first tested the possible involvement of these proteins in C. difficile H_2_O_2_ resistance. Using disk diffusion assays, we did not observe any difference in the growth inhibition area for the *ΔrevRbr1*/*2*, the *ΔrevRbr1/2-ΔfdpF*, and the Δ*revRbr1/2*-Δ*fdpF*-*fdpA* mutants compared to the WT strain in the presence of H_2_O_2_ (see Fig. S6B). These results suggest either that these H_2_O_2_-reductase activities have no physiological role in the detoxification of H_2_O_2_ in C. difficile or that the existence of other enzymes scavenging H_2_O_2_ prevents to observe a phenotype in our conditions.

### Involvement of revRbrs and FDPs in *C. difficile* low O_2_ tolerance.

**FIG 5:**
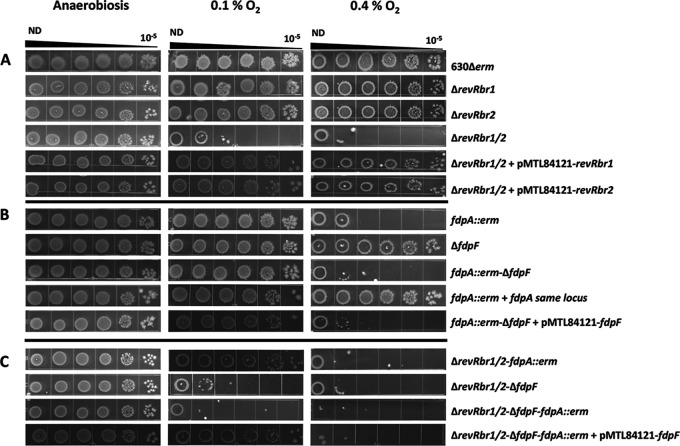
Role of revRbrs and FDPs in low O_2_ tension tolerance. Serial dilutions of the 630Δ*erm*, the different mutants, and the complemented strains were spotted on TY taurocholate plates. Plates were then incubated 64 h either in anaerobic atmosphere (control) or in the presence of 0.1 or 0.4% O_2_. These pictures are representative of four and three independent experiments for the mutant and complemented strains, respectively (see also Fig. S7 and S8). (A) Role of revRbrs in low O_2_ tension tolerance; (B) role of FDPs in low O_2_ tension tolerance; (C) additive O_2_-reductase activities of FDPs and revRbrs.

Since both revRbrs and both FDPs act as NADH dependent O_2_-reductases (Table 1), we assessed the ability of the different mutants to tolerate low O_2_ tensions. For this purpose, we incubated tryptone-yeast extract (TY) plates spotted with serial dilutions of each strain in anaerobiosis or in the presence of different low O_2_ tensions (0.1 or 0.4%). No difference in growth between the WT strain and the mutants was observed in anaerobiosis (Fig. 5). Similarly, we did not detect any growth defect for the Δ*revRbr1*, Δ*revRbr2*, and Δ*fdpF* single mutants compared to the WT strain when exposed to O_2_ tensions up to 0.4% (Fig. 5A and B). In contrast, there was a growth defect for the *fdpA* mutant under these conditions, whereas no differences were observed in the presence of lower O_2_ tensions (i.e., 0.1 and 0.2%) (Fig. 5B; see also Fig. S7A). We were not able to complement this reduced O_2_ tolerance by expressing *fdpA* on a plasmid either from its own promoter (both σ^A^ and σ^B^ dependent promoters in pMTL84121 plasmid) or from an anhydrotetracycline (aTc)-inducible promoter (P_tet_) (pDIA6103 plasmid) (see Fig. S7B). Of note, overexpression of *fdpA* in the presence of 100 ng ml^−1^ of aTc resulted in a growth defect even in anaerobiosis, suggesting that the overexpression of *fdpA* is toxic for C. difficile. On the other hand, the growth defect observed for the *fdpA* mutant in the presence of 0.4% O_2_ was restored when the *fdpA* disrupted by the clostron insertion was replaced by a wild-type copy of *fdpA* using the ACE technique (Fig. 5B). In addition, we observed the same growth defect for a Δ*fdpA* mutant generated by ACE in the presence of 0.4% O_2_ (see Fig. S7B). Altogether, these results indicate that FdpA plays a role in low O_2_ tolerance in C. difficile. Strikingly, deletion of both revRbr-encoding genes strongly affected the growth compared to each single mutant and the WT strain in the presence of 0.1 or 0.4% O_2_ (Fig. 5A). The growth of this double mutant was fully restored when we expressed either the *revRbr1* or the *revRbr2* gene under the control of their own promoter, indicating a functional redundancy between both revRbrs in their ability to reduce O_2_ and confirming a crucial role of revRbrs in O_2_ tolerance in C. difficile. In contrast to revRbrs, the inactivation of both FDP-encoding genes only resulted in a slight but reproducible decreased O_2_ tolerance compared to the *fdpA* single mutant at 0.4% (Fig. 5B; see also Fig. S7C). In addition, the introduction of pMTL84121-Pσ^B^-*fdpF* did not increase the growth of the *fdpA*-Δ*fdpF* mutant, indicating that FdpF cannot replace FdpA to restore O_2_ tolerance at 0.4% (Fig. 5B).

10.1128/mBio.01559-20.7 FIG S7 Involvement of FDPs in low O_2_ tension tolerance. (A) Serial dilutions of the 630Δ*erm*, *the fdpA*::*erm*, Δ*fdpF*, and *fdpA*::*erm*-Δ*fdpF* mutants were spotted onto TY taurocholate plates. Plates were then incubated 64 h either in anaerobic atmosphere (control) or in presence of 0.2% O_2_. These pictures are representative of three independent experiments. (B) Serial dilutions of the 630Δ*erm* mutant, the two different *fdpA* mutants (*fdpA*::*erm* clostron and Δ*fdpA* mutants), and the complemented strains were spotted onto TY taurocholate plates. Plates were then incubated 64 h either in an anaerobic atmosphere (control) or in the presence of 0.4% O_2_. The expression inducer aTc was added directly to the TY taurocholate plates. These pictures are representative of two independent experiments. (C) Different replicates of low oxygen tolerance assay with the 630Δ*erm* strain, the different mutants inactivated for FDPs-encoding genes, and the complemented strains. Download FIG S7, TIF file, 1.9 MB. Copyright © 2020 Kint et al. 2020 Kint et al. This content is distributed under the terms of the Creative Commons Attribution 4.0 International license.

10.1128/mBio.01559-20.8 FIG S8 Additive effect of FDPs and revRbrs in low O_2_ tension tolerance. Different replicates of low oxygen tolerance assay with the different mutants inactivated for FDPs- and revRbrs-encoding genes and the complemented strains were performed. Download FIG S8, TIF file, 2.0 MB. Copyright © 2020 Kint et al. 2020 Kint et al. This content is distributed under the terms of the Creative Commons Attribution 4.0 International license.

To determine whether the O_2_-reductase activities of both FDPs and revRbrs could be additive, we exposed the Δ*revRbr1/2-*Δ*fdpF* and the Δ*revRbr1/2*-*fdpA* triple mutants, as well as the quadruple mutant, to low O_2_ tensions. Inactivation of *fdpF* did not decrease the O_2_ tolerance of the Δ*revRbr1/2* double mutant whatever the O_2_ tension tested (Fig. 5C; see also Fig. S8). Interestingly, inactivation of *fdpA* increased the O_2_ tolerance of the Δ*revRbr1/2* double mutant when exposed to 0.1% but not 0.4% O_2_. This effect was FdpF dependent since the quadruple mutant (Δ*revRbr1/2-ΔfdpF-fdpA*::*erm*) was less tolerant than the Δ*revRbr1/2* double mutant and both triple mutants to O_2_ from concentrations as low as 0.1% (Fig. 5C; see also Fig. S8). Remarkably, we noticed that the introduction of pMTL84121-Pσ^B^-*fdpF* restored the O_2_ tolerance observed in the Δ*revRbr1/2-fdpA*::*erm* triple mutant in the presence of 0.1% O_2_, confirming the role of FdpF in O_2_ tolerance at extremely low O_2_ tension (Fig. 5C). In addition, at 0.4% O_2_, no colony was observed for the quadruple mutant while both triple mutants were able to grow when they were non-diluted (Fig. 5C; see also Fig. S8). From these results, we conclude that O_2_-reductase activities of both FDPs and revRbrs are additive, and we propose that FdpF plays a minor but visible role in O_2_ tolerance under these conditions. Altogether, these results demonstrate the role of both revRbrs and FDPs in O_2_ tolerance mechanisms in C. difficile.

## DISCUSSION

In the present study, we identified two revRbrs and two FDPs as the O_2_-reductases in C. difficile. We showed that these enzymes are important for O_2_ tolerance of this bacterium and that their activities are additive. Indeed, a quadruple mutant inactivated for all genes encoding these proteins has a strong growth defect at 0.1% O_2_ and is unable to grow in the presence of 0.4% O_2_. This demonstrates for the first time the physiological role of these two classes of enzymes in the ability of a strict anaerobe to tolerate low physiological O_2_ tensions (<0.5%). It is worth noting that O_2_ tolerance varies among the different C. difficile ribotypes. Indeed, whereas the survival of R20291 (ribotype 027) or 5235 (ribotype 078) vegetative cells sharply decreases after 3 h of air exposure, vegetative cells of 630Δ*erm* strain (ribotype 012) are still detectable after 24 h (54). Moreover, the R20291 strain has a decreased O_2_ tolerance compared to the 630Δ*erm* strain in the presence of 0.1 and 0.4% O_2_ (17). However, in both 630Δ*erm* and R20291 backgrounds, this low O_2_ tolerance process depends on the alternative sigma factor involved in general stress response, σ^B^, which is involved in transcription of *fdpA*, *fdpF*, *revRbr1*, and *revRbr2* (17; this study). Accordingly, both revRbr- and FDP-encoding genes harbor a σ^B^-consensus sequence in their promoter, and the phenotype of the quadruple mutant at low O_2_ tension phenocopies that of the *sigB* mutant. However, while the transcription of *fdpF*, *revRbr1*, or *revRbr2* appears to be strictly σ^B^ dependent (Fig. 1) (39), *fdpA* is transcribed from two promoters depending on σ^A^ and σ^B^. Of note, the expression of *fdpA*, *fdpF*, *revRbr1*, or *revRbr2* is not induced by amoxicillin and clindamycin exposure, two antibiotics known to promote CDI through the disruption of a healthy microbiota that results in an increased O_2_ level in the gut (see below) (55). On the other hand, the expression of these genes is upregulated upon brief aeration of C. difficile, whereas no induction is observed after a short exposure to 5% O_2_ or when grown in microaerophilic conditions (2% O_2_) (15, 55, 56). These discrepancies could be linked to the differences in stress exposure conditions. Further studies are required to elucidate the exact role of air or low O_2_ exposure on the expression of genes encoding revRbrs and FDPs and whether this effect is mediated by σ^B^. Interestingly, homologs of revRbr1/2 (Rbr3A/B) and of two class A FDPs (FprA1/2) are present in C. acetobutylicum, and the expression of the corresponding genes is induced upon 5% O_2_ flushing (21, 57, 58). In this bacterium lacking σ^B^, the expression of *rbr3A/B* and *fprA1/2* is negatively controlled by the PerR repressor (59, 60), and a *perR* mutant has increased O_2_ consumption, aerotolerance, and ROS resistance (60). In C. difficile, a PerR-encoding gene cluster is present. This cluster contains genes encoding a canonical rubrerythrin (*CD0825*) and a desulfoferrodoxin (*CD0827*) (a superoxide reductase) that are homologs to PerR targets in C. acetobutylicum (59). Using the consensus defined in C. acetobutylicum, we failed to identify an identical PerR box in the promoter region of *fdp* and *revRbr* genes but also upstream the *CD0825* operon. The role of PerR in the physiology of C. difficile and in the control of *fdp* and *revRbr* genes remains to be studied.

We also demonstrated that both purified revRbrs reduce O_2_ and H_2_O_2_ into H_2_O, although H_2_O_2_ is the preferred substrate, as observed for revRbrs in C. acetobutylicum (20). On the other hand, FdpA only acts as NADH-linked O_2_-reductase *in vitro.* Although some FDPs have been shown to scavenge both NO and O_2_ (32, 45), FdpA and FdpF lack this dual activity (see Fig. S8) (35). In C. acetobutylicum, O_2_-reductase activity of class A FDPs FprA1/2 are 100 times greater than those of Rbr3A/B (20, 21). In our conditions, O_2_-reductase activities of both revRbrs and FdpA are rather similar and 10-fold lower than the one described for FdpF (between 1.2 and 2 s^−1^ compared to 16 s^−1^) (35). However, in FdpF, all the domains involved in the electron transfer from NADH to O_2_ are present, while revRbrs and FdpA need intermediate proteins to perform such a transfer. Since the natural electron transfer proteins associated with revRbrs and FdpA remain unknown in C. difficile, our assays were performed with Rd-D and NROR from E. coli that most probably result in a lower O_2_-reductase activity. Therefore, the activities determined in these experimental conditions may be considerably underestimated. C. difficile 630 has no genes encoding Rds, as also observed in many organisms having FDPs and in which the electron donors of these enzymes remain also to be determined (24).

Even if the enzymatic properties of revRbrs and FDPs from several microorganisms have been characterized, less is known about their physiological role, especially in the ability of anaerobes to deal with O_2_ (24, 61). In C. difficile, the relative importance of both revRbrs and FDPs in O_2_ tolerance seems to be variable. Indeed, only the *fdpA* single mutant showed a reduced O_2_ tolerance, while we need to inactivate both revRbrs-encoding genes to observe a growth defect in the presence of low O_2_ tensions. This clearly indicates a functional redundancy of revRbrs sharing both a high level of similarity and a common control of their production by σ^B^. On the other hand, the double mutant Δ*revRbr1/2* showed a strongly reduced tolerance to 0.1% O_2_, highlighting the crucial role of these proteins in the growth of C. difficile at very low O_2_ tensions. Surprisingly, *fdpA* inactivation in the Δ*revRbr1/2* mutant increased the tolerance at 0.1% O_2_. Although the underlying mechanism responsible for this higher tolerance is not characterized, this effect is dependent on FdpF, since its inactivation completely abolished the growth whatever the O_2_ concentration tested (Fig. 5). Further investigations are needed concerning the expression or the activity of FdpF in different genetic backgrounds and oxygenation conditions. While the two revRbrs and FdpF also harbor significant H_2_O_2_-reductase activities *in vitro* (35), inactivation of these genes does not result in increased H_2_O_2_ sensitivity, strongly suggesting that other enzymes efficiently scavenging H_2_O_2_ are present in C. difficile. Three catalases and a peroxidase (CotE) are present in C. difficile but only produced during sporulation (33, 62). However, a secreted glutamate dehydrogenase has been shown to participate in resistance to H_2_O_2_ (63). In addition, other proteins such as a canonical Rbr (CD0825) and a bacterioferritin comigratory protein (CD1822), that are homologs to proteins with peroxidase activity in other organisms (61) might contribute to protect C. difficile vegetative cells from H_2_O_2_ stress. Further work is needed to characterize all the pathways involved in H_2_O_2_ protection and to demonstrate the possible physiological role of the revRbrs and FdpF in H_2_O_2_ detoxification.

Previous studies have shown that NO-specific FDPs play a role in the protection of bacteria from NO-related killing within macrophages (64, 65). However, no studies have linked O_2_-specific FDPs and revRbrs with virulence or colonization. Several recent studies demonstrate that antibiotic administration, a CDI risk factor, increased the O_2_ level within the gut by modifying the microbiota. Indeed, antibiotherapy notably depletes clostridia that produce butyrate, a compound directly consumed by enterocytes through O_2_ conversion to CO_2_ (14, 66). Moreover, some vegetative cells have been found associated with the mucus (67–69), a place in which the O_2_ level is higher than in the lumen (11, 70). Thus, C. difficile vegetative cells cope with O_2_ tensions during the colonization process and, in such nonstrict anaerobic environments, the ability to tolerate small amounts of O_2_ appears to be essential (55). Accordingly, both *sigB* and *iscS2* mutants, affected in their ability to tolerate low O_2_ tensions, have been shown to be impaired in colonization in axenic mice (16, 17). Since both revRbrs and FDPs play a crucial role in C. difficile low O_2_ tolerance, further studies are needed to show that these proteins are important during the colonization process.

## MATERIALS AND METHODS

### Bacterial strains and growth conditions.

The C. difficile strains and plasmids used in this study are listed in Table S1 in the supplemental material. C. difficile strains were grown anaerobically (5% H_2_, 5% CO_2_, 90% N_2_) in TY (Bacto tryptone, 30 g liter^−1^, yeast extract, 20 g liter^−1^ [pH 7.4]), in Pep-M (71), or in C. difficile minimal medium (CDMM) (72). For solid media, agar was added to a final concentration of 17 g liter^−1^. When necessary, cefoxitin (Cfx; 25 μg ml^−1^), thiamphenicol (Tm; 15 μg ml^−1^), or erythromycin (Erm; 2.5 μg ml^−1^) were added to C. difficile cultures. E. coli strains were grown in LB broth or in M9 minimal medium supplemented with FeSO_4_ to a final concentration of 200 μM. When indicated, ampicillin (100 μg ml^−1^), chloramphenicol (15 μg ml^−1^), or kanamycin (50 μg ml^−1^) was added to the culture medium.

10.1128/mBio.01559-20.9 TABLE S1 Strains, plasmids, and oligonucleotides used in this study. (A) Strains and plasmids. (B) Oligonucleotides. Download Table S1, DOC file, 0.1 MB. Copyright © 2020 Kint et al. 2020 Kint et al. This content is distributed under the terms of the Creative Commons Attribution 4.0 International license.

### Construction of *C. difficile* strains.

The clostron gene knockout system (73, 74) was used to inactivate the *fdpA* (*CD1157*) gene, yielding the insertional mutant strain 630Δ*erm fdpA*::*erm* (CDIP588). We designed primers to retarget the group II intron of pMTL007-CE5 to insert it into the *fdpA* gene in sense orientation after nucleotide 225 in the coding sequence (see Table S1). The PCR product generated by overlap extension was cloned between the HindIII and BsrG1 sites of pMTL007-CE5 to obtain pDIA6374. C. difficile transconjugants obtained with E. coli HB101(RP4) containing pDIA6374 were selected on brain heart infusion (BHI) agar containing Tm and Cfx and then plated on BHI agar containing Erm. PCRs on transconjugants chromosomal DNA were performed to verify the integration of the intron into the *fdpA* gene and the splicing of the group I intron from the group II intron after integration.

The Δ*revRbr1* (Δ*CD1474*), Δ*revRbr2* (Δ*CD1524*), Δ*fdpA* (Δ*CD1157*), and Δ*fdpF* (Δ*CD1623*) knockout mutants were obtained using the *codA*-mediated allele exchange method (75, 76). Then, 1.5-kb fragments located up- and downstream of these genes were PCR amplified from 630Δ*erm* genomic DNA using the pairs IMV917/NK70 and NK71/IMV918, IMV919/NK64 and NK65/IMV920, CF80/CF81 and NK180/CF83, and IMV914/NK58 and NK59/IMV915 (Table S1B) for Δ*revRbr1*, Δ*revRbr2*, Δ*fdpA*, and Δ*fdpF*, respectively. Purified PCR fragments were then introduced into the pMTLSC7315 plasmid using a Gibson Assembly master mix (Biolabs). The sequences of the resulting plasmids were verified by sequencing. The plasmids obtained introduced in HB101(RP4) E. coli strain were transferred by conjugation into the C. difficile 630Δ*erm* strain. Transconjugants were selected on BHI plates supplemented with Tm and C. difficile selective supplement (SR0096; Oxoid). Isolation of faster growing single-crossover integrants was performed by serial restreaking on BHI plates containing Cfx and Tm. Single-crossover integrants were then restreaked on CDMM plates supplemented with fluorocytosine (50 μg ml^−1^), allowing the isolation of double-crossover events. After confirmation of plasmid loss (Tm-sensitive clones), the presence of the expected deletion in clones was checked by PCR. Steps were repeated in each different mutant in order to generate C. difficile multi mutants (see Table S1A).

### Complementation of the different mutants.

To complement *revRbr1*, *revRbr2*, and *fdpF*, the promoter region and the open reading frame of the corresponding gene were amplified by PCR using oligonucleotide pairs CF115/CF116, CF113/CF114, and IMV699/IMV700, respectively. The PCR products were cloned into pMTL84121 to produce pDIA6538, pDIA6537, and pDIA6388 (see Table S1A). For *fdpA*, the region encompassing both σ^A^- and σ^B^-dependent promoters and the open reading frame was amplified by NK259/NK260 and inserted by Gibson Assembly into pMTL84121 amplified by inverse PCR with NK261/NK262 to yield pDIA6870. To express *fdpA* under the control of the inducible P_tet_ promoter, the open reading frame was amplified by PCR using oligonucleotides IMV978 and IMV979. The PCR product was cloned into pDIA6103 (37) to produce pDIA6806. E. coli HB101(RP4) strain containing each plasmid was mated with single or double mutants (see Table S1). To complement the *fdpA* mutant at the same locus, the clostron was removed by the chromosomal introduction of a *fdpA* wild-type copy using ACE. First, a PCR fragment encompassing *fdpA* and 1.5-kb fragments located upstream and downstream of *fdpA* gene was amplified from 630Δ*erm* genomic DNA using primers CF80/CF83. This fragment was then introduced into pMTLSC7315 by Gibson Assembly, yielding pDIA6956. The resulting plasmid was transferred from E. coli HB101(RP4) to the *fdpA*::*erm* mutant by mating. Selection of transconjugants, isolation of faster growing single-crossover integrants, and isolation of double-crossover events were performed as described above. After confirmation of plasmid loss, the presence of the *fdpA* gene in lieu of the intron was checked by PCR.

### Oxygen and H_2_O_2_ stress tolerance assays.

For O_2_ tolerance assays, 5 μl of different serial dilutions (from 10^0^ to 10^−5^) of C. difficile strains grown for 7 h in TY were spotted on TY plates containing 0.05% taurocholate. Plates were then incubated in the presence of different O_2_ tensions or in anaerobiosis in a microaerophilic workstation from Baker Ruskinn. When needed, aTc was added to the plate to induce *fdpA* expression. The last dilution allowing growth was recorded after incubation at 37°C for 64 h. Disk diffusion assays were conducted as follows. Cultures of C. difficile strains grown in Pep-M medium were plated on calibrated Pep-M agar. A sterile 6-mm paper disk was placed on the agar surface, and 4 μl of 1 M hydrogen peroxide (H_2_O_2_) was added to the disk. The diameter of the growth inhibition area was measured after 24 h of incubation at 37°C.

### Transcriptional SNAP*^Cd^* fusions.

To construct transcriptional fusions, we used pFT47, a plasmid containing the SNAP*^Cd^* gene (38). A fragment containing either the complete promoter region of *fdpA* (positions −308 to +114 from the start codon) or only the σ^B^-dependent promoter region (−104 to +114) was amplified using genomic DNA and the primer pairs IMV949/IMV950 and IMV976/IMV950, respectively. The DNA fragments were inserted between the EcoRI and XhoI sites of pFT47 to obtain pDIA6517 and PDIA6669, respectively. To construct the *revRbr2*- or *fdpF*-transcriptional SNAP*^Cd^* fusion, the promoter region (−190 to +30 or −202 to +14 from the TSS) was amplified using genomic DNA and primer pairs CF23/CF24 or CF21/CF22, respectively. The DNA fragments were inserted into pFT47 between the EcoRI and XhoI sites to obtain pDIA6459 and pDIA6458. The plasmids introduced into E. coli HB101(RP4) were then transferred by conjugation into C. difficile 630Δ*erm* strain and the *sigB* mutant (see Table S1).

### Fluorescence microscopy and image analysis.

To monitor the expression of the different transcriptional fusions, the strains were grown for 16 h in BHI. SNAP labeling and fluorescence microscopy were performed as previously described (38). The images were taken with exposure times of 200 ms for autofluorescence and 500 ms for SNAP. Cells were observed on a Nikon Eclipse TI-E microscope 60× objective and captured with a CoolSNAP HQ2 Camera. For quantification of the SNAP-TMR Star signal resulting from transcriptional fusions, the pixel intensity was measured and corrected by subtracting the average pixel intensity of the background. Images were analyzed using ImageJ.

### Protein production, purification, and quaternary structure determination.

The coding region of *revRbr1* and *revRbr2* were PCR amplified using genomic DNA and primer pairs IMV970/IMV971 or IMV968/IMV969 to produce a 540-bp PCR product that was cloned into pET20 (Novagen), yielding pDIA6635 or pDIA6671. The amino acid sequence of FdpA was used to synthesize its encoding gene (GenScript, Inc., USA) using codon optimized for expression in E. coli, which was cloned into a pET24a plasmid. After verification by sequencing, the plasmids were introduced in E. coli BL21(DE3)Gold. Strains overexpressing *revRbr1*, *revRbr2*, or *fdpA* were grown aerobically at 37°C at 150 rpm in M9 minimal medium, with 20 mM glucose, 0.1 mM FeSO_4_, and ampicillin (for *revRbr1* and *revRbr2*) or kanamycin (for *fdpA*). When the optical density at 600 nm reached 0.4, 0.1 mM FeSO_4_ was added, and gene expression was induced by 1 mM IPTG (isopropyl-β-d-thiogalactopyranoside) for both *revRbrs* and 0.1 mM IPTG for *fdpA*. After 6 h of growth at 30°C, the cells were harvested by centrifugation, resuspended in a buffer containing 50 mM Tris-HCl (pH 7.5), and stored at –20°C. Cells were disrupted by at least three cycles in a French press apparatus at 16,000 lb/in^2^ (Thermo) in the presence of DNase (Applichem). The crude extract was cleared by low-speed centrifugation at 25,000 × *g* for 25 min and then at 138,000 × *g* for 90 min at 4°C to remove cell debris and the membrane fraction, respectively. The soluble extract was dialyzed overnight at 4°C against buffer A (20 mM Tris-HCl [pH 7.5], 18% glycerol) and subsequently loaded onto a Q-Sepharose Fast Flow column (65 ml; GE Healthcare) previously equilibrated with buffer A. Proteins were eluted with a linear gradient from buffer A to buffer B (buffer A containing 500 mM NaCl). The eluted fractions were monitored throughout the purification process by SDS-PAGE and UV-visible spectroscopy. Fractions containing the desired protein were pooled and concentrated. For both revRbrs, the concentrated fraction was then loaded onto a size exclusion Superdex S75 column (330 ml; GE Healthcare) equilibrated with buffer A containing 150 mM NaCl. The fractions containing the protein were pooled and concentrated. For revRbr1, the last fraction was loaded onto a Fractogel column (20 ml; Merck Millipore) previously equilibrated with buffer A and eluted with a linear gradient from buffer A to buffer B. After purification, FdpA was incubated overnight at 4°C in the presence of 1 mM FMN and subsequently applied to a PD-10 desalting column to eliminate unbounded FMN. Fractions containing purified proteins were verified by SDS-PAGE (see Fig. S3A).

The quaternary structures of the proteins were determined by size exclusion chromatography. Proteins were loaded onto a 25-ml Superdex S200 10/300 GL column (GE Healthcare) previously equilibrated with buffer A containing 150 mM NaCl. A mixture containing tyroglobulin (669 kDa), apoferritin (443 kDa), β-amylase (200 kDa), alcohol dehydrogenase (150 kDa), albumin (66 kDa), carbonic anhydrase (29 kDa), and dextran blue (2000 kDa) as a void volume marker was used as the standard (see Fig. S3B).

### Protein, metal, and flavin quantification.

Purified protein samples were quantified using a bicinchoninic acid kit (Thermo) and bovine serum albumin as the standard. The iron content was determined by the phenanthroline colorimetric method. Protein samples were incubated for 15 min with 1 M HCl and for further 30 min with 10% trichloroacetic acid at room temperature, centrifuged at 5,000 × g for 20 min, and neutralized by the addition of 15% ammonium acetate. Samples were then incubated with 10% hydroxylamine and 0.3% 1-10-phenantrhroline. The absorbance spectrum was measured, and the iron content was quantified by using ɛ_510_ = 11.2 mM^−1^ cm^−1^. The flavin type and content were determined as previously described (35).

### Spectroscopic methods.

UV-visible spectra were obtained in a Perkin-Elmer Lambda 35 spectrophotometer. Electron paramagnetic resonance (EPR) spectroscopy was performed using a Bruker EMX spectrometer equipped with an Oxford Instruments ESR-900 continuous-flow helium cryostat and a high-sensitivity perpendicular mode rectangular cavity. Protein samples were prepared aerobically to final concentrations of 1 mM (FdpA and revRbr2) or 600 μM (revRbr1). Partially reduced samples were also prepared anaerobically by incubation with 1 equivalent of menadiol.

### Redox titrations.

The reduction potentials of the FMN from FdpA and of the Rd domain from the revRbrs were determined by redox titrations monitored by visible absorption spectroscopy in a Shimadzu UV-1603 spectrophotometer. Protein samples at a 30 μM final concentration in buffer C (50 mM Tris-HCl [pH 7.5] and 18% glycerol) were titrated inside an anaerobic chamber (Coy Lab Products) by stepwise addition of a buffered sodium dithionite solution in the presence of a mixture of redox mediators (0.5 μM each): *N*,*N*-dimethyl-*p*-phenylenediamine (E′° = +340 mV), 1,2 naphtoquinone-4-sulfonic acid (E′° = +215 mV), 1,2 naphtoquinone (E′° = +180 mV), trimethylhidroquinone (E′° = +115 mV), phenazine methosulfate (E′° = +80 mV), 1, 4 naphtoquinone (E′° = +60 mV), phenazine ethosulfate (E′° = +55 mV), 5-hydroxy-1,4-naphtoquinone (E′° = +30 mV), duroquinone (E′° = +5mV), menadione (E′° = +0 mV), plumbagin (E′° = –40 mV), resorufin (E′° = –51 mV), indigo trisulfonate (E′° = –70 mV), indigo disulfonate (E′° = –110 mV), phenazine (E′° = –125 mV), 2,5-hydroxy-*p*-benzoquinone (E′° = –130 mV), 2-hydroxy-1,4-naphtoquinone (E′° = –152 mV), anthraquinone-2-sulfonate (E′° = –225 mV), phenosafranine (E′° = –255 mV), safranine (E′° = –280 mV), and neutral Red (E′° = –325 mV). A combined Pt electrode (Ag/AgCl in 3.5 M KCl, as a reference) was used and calibrated at 23°C against a saturated quinhydrone solution (pH 7). The reduction potentials are reported in relation to the standard hydrogen electrode. The reduction potentials determined for revRbrs and FdpA were calculated by adjusting a Nernst equation to the experimental data obtained by UV-visible spectroscopy. In all cases, the reduction potential of the diiron sites could not be determined since they have very low molar absorptivities. For the revRbrs, the absorption values obtained at 490 nm (typical of the oxidized Rd-like site) were normalized in relation to the full oxidized protein, and a theoretical curve of the oxidized population was fitted using a SciLab routine and applying a Nernst equation for a one-electron transfer process. The populations were calculated as follows:

$$E=E^{0}+\mathrm{RT}\ln\left(Q\right),$$

where *Q* is calculated as follows:

$$Q=\frac{[\mathrm{Rd}]\mathrm{Ox}}{[\mathrm{Rd}]\mathrm{Red}}$$

Therefore,

$$[\mathrm{Rd}]\mathrm{Red}=\frac{10^{\frac{E0-E}{Y}}}{1+10^{\frac{E0-E}{Y}}}$$

[Rd]Ox=1−[Rd]Red

where

$$Y=2.303\frac{\mathrm{RT}}{\mathrm{nF}}$$

where *R* = 8.314 J K^−1^ mol^−1^, *T* = 298.15 K, *n* = 1 electron, and *F* = 96485 C mol^−1^.

The theoretical model and curve adjustment were performed using Scilab 6.0.2. The *E*_0_ for the rubredoxin center of the two revRbrs was determined from the fit. For FdpA, the FMN moiety is the only contributor for the visible spectrum and undergoes two consecutive redox reactions. The reduction potentials were determined as for the revRbrs, using the appropriate Nernst equation for two sequential monoelectronic processes and taking into consideration the molar absorptions of the oxidized and semireduced forms of the flavin.

$${\text{FMN}}_{\text{0X}}\overset{E_{\text{01}}}{↔}{\text{FMN}}_{\text{SQ}}\overset{E_{\text{02}}}{↔}{\text{FMN}}_{\text{Red}}$$

Therefore, the experimental data measured at 450 nm were normalized and fitted considering the contribution of both the oxidized and the semireduced (semiquinone [SQ]) forms of FMN multiplied by the respective molar absorption coefficients at this wavelength. The populations were calculated as follows:

$$\left[\mathrm{FMN}\right]SQ=\frac{10^{\frac{E01-E}{Y}}}{1+10^{\frac{E01-E}{Y}}+10^{\frac{E01+E02-2*E}{Y}}}$$

$$[\mathrm{FMN}]\mathrm{Red}=\frac{10^{\frac{E01+E02-2*E}{Y}}}{1+10^{\frac{E01-E}{Y}}+10^{\frac{E01+E02-2*E}{Y}}}$$

[FMN]Ox=1−[FMN]Red−[FMN]SQ,

where

$$Y=2.303\frac{\mathrm{RT}}{\mathrm{nF}}$$

and where *R* = 8.314 JK^−1^ mol^−1^, *T* = 298.15 K, *n* = 1 electron, and *F* = 96485 C mol^−1^.

As before, the theoretical model and curve adjustment were performed using Scilab 6.0.2. The *E*_01_ and *E*_02_ were determined from the fit.

### Spectrophotometric measurement of the H_2_O_2_-reductase activity.

Because the physiological electron donor to the C. difficile revRbrs and FdpA is unknown, we used an artificial electron-donating system: a mixture of the Rd domain (Rd-D) of the E. coli FDP (flavoRd) and of the flavoRd reductase (NROR, gene ID 947088, E. coli strain K-12). The E. coli proteins were overexpressed and purified as previously described (50). The enzymatic activity for H_2_O_2_ was determined by UV-visible spectroscopy, inside an anaerobic chamber (Coy Lab Products). The assays were performed in buffer C. The reaction was monitored at 340 nm, determining the NADH consumption (ε_340_ = 6,220 mM^−1 ^cm^−1^). A mixture of buffer containing 200 μM NADH, NROR (2.5 or 5 μM for the revRbrs or FdpA assays, respectively), and Rd-D (2.5 or 5 μM for revRbrs or FdpA assays, respectively) was used. Different amounts of NROR and Rd-D were tested and optimized in combination with each enzyme in order to ensure that the reaction rates were maximized. The reaction was initiated by the addition of H_2_O_2_. Concentrations of revRbr1, revRbr2, or FdpA were 0.1, 0.03, and 1 μM, respectively. Different amounts of H_2_O_2_ were used for each of the three enzymes to evaluate the dependence of the rates with the amount of substrate. No significant differences were verified within the range of concentrations used (10 to 150 μM). This indicates that, if these enzymes follow a Michalis-Menten behavior, the *K_m_* value is low and therefore not covered by the range used. This also indicates that all the assays are in Vmax conditions. The calculated rates (s^−1^) presented in Table 1 were calculated subtracting the experimental slope (μM s^−1^) before and after the addition of H_2_O_2_, divided by the protein concentration (μM).

### Measurement of O_2_-reductase activity.

The O_2_-reductase activity of the proteins was measured amperometrically with a Clark-type electrode selective for O_2_ (Oxygraph-2K; Oroboros Instruments, Innsbruck, Austria). The assays were performed in buffer C. The O_2_-reductase activity was evaluated at 25°C in air equilibrated buffer (∼260 μM O_2_) in the presence of 5 mM NADH, NROR (2.5 μM), and Rd-D (2.5 μM). Different amounts of NROR and Rd-D were tested and optimized in combination with each enzyme in order to ensure that the reaction rates were maximized. The reaction was initiated by addition of revRbr2 (0.1 μM), revRbr1 (0.1 μM), or FdpA (1 μM). Assays were performed in the presence of catalase (640 nM). The calculated rates (s^–1^) presented in Table 1 were calculated subtracting the experimental slope (μM s^−1^) before and after the addition of each enzyme, divided by the protein concentration (μM).

### Measurement of NO-reductase activity.

The NO-reductase activity of FdpA was measured amperometrically with a Clark-type electrode selective for NO (ISO-NOP; World Precision Instruments, Sarasota, FL). The assays were performed in buffer C. The NO-reductase activity was evaluated at 25°C in degassed buffer in the presence of an O_2_-scavenging system (10 mM glucose, 375 nM glucose oxidase, and 750 nM catalase), NROR (2.5 μM), and Rd-D (2.5 μM). Sequential additions of NO (up to 12 μM) were followed by the addition of 5 mM NADH, and the reaction was initiated by the addition of FdpA (0.1 μM). Stock solutions of 1.91 mM NO were prepared by saturating a degassed buffer C in a rubber seal-capped flask with pure NO gas (Air Liquide) at 1 atm on ice: gaseous NO was flushed through a 5 mM NaOH trap to remove higher N-oxides, and a second trap with deionized water to remove aerosols. After this, the solution was allowed to equilibrate at room temperature.
