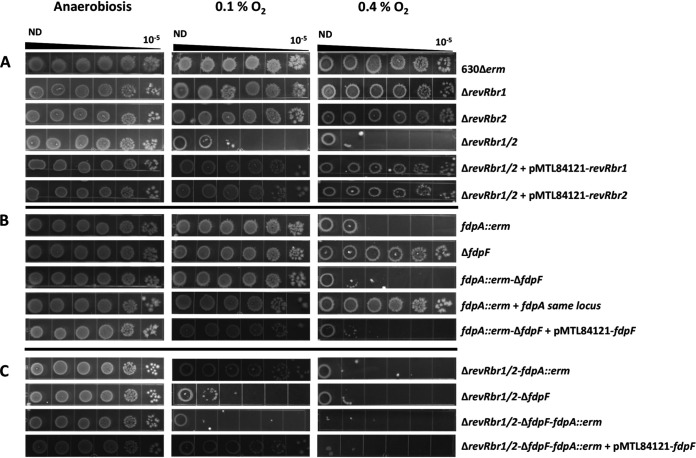# Erratum for Kint et al., “How the Anaerobic Enteropathogen Clostridioides difficile Tolerates Low O_2_ Tensions”

**DOI:** 10.1128/mBio.02678-20

**Published:** 2020-10-27

**Authors:** Nicolas Kint, Carolina Alves Feliciano, Maria C. Martins, Claire Morvan, Susana F. Fernandes, Filipe Folgosa, Bruno Dupuy, Miguel Texeira, Isabelle Martin-Verstraete

**Affiliations:** a Laboratoire Pathogenèses des Bactéries Anaérobies, Institut Pasteur, UMR CNRS 2001, Université de Paris, Paris, France; b Instituto de Tecnologia Química e Biológica António Xavier, Universidade Nova de Lisboa, Oeiras, Portugal; c Institut Universitaire de France

## ERRATUM

Volume 11, no. 5, e01559-20, 2020. https://doi.org/10.1128/mBio.01559-20. On PDF page 8, Fig. 5 should be replaced by the figure below. In the supplemental material, Fig. S7 has been replaced online. The figure legends are not modified. These changes do not change the conclusions of the paper.

**Figure 1:**