# Supplementary material for: How the Anaerobic Enteropathogen Clostridioides difficile Tolerates Low O2 Tensions
Source: mBio. 2020 Sep 8;11(5):e01559-20. doi: 10.1128/mBio.01559-20 (PMC7482061; doi:10.1128/mBio.01559-20)
Supplement: TABLE S1 [file mBio.01559-20-st001.doc]

**Table S1A: Strains and plasmids used in this study.**

| **Strains** | **Genotypes** | **Origins** |
| --- | --- | --- |
| ***E. coli*** |  |  |
| 10-beta | F^-^ *mcr*A D(*mrr-hsd*RMS-*mcr*BC) f80*lac*ZDM15 D*lac*X74 *deo*R, *rec*A1 *ara*D139 D(*ara-leu*)7697 *gal*K *rps*L(StrR) *end*A1 *nup*G | NEB® |
| HB101(RP4) | *supE*44 *aa*14 *galK*2 *lacY*1 ∆(*gpt-proA*) 62 *rpsL*20 (Str^R^)*xyl-5 mtl-1 recA*13 ∆(*mcrC-mrr*) *hsdS*_B_(r_B_^-^m_B_^-^) RP4 (Tra^+^ IncP Ap^R^ Km^R^ Tc^R^) | Laboratory stock |
| BL21(DE3) |  | Laboratory stock |
| ECO831 | BL21 (DE3) pET20-*revRbr1* | pDIA6635→ BL21(DE3) |
| ECO860 | BL21 (DE3) pET20-*revRbr2* | pDIA6671→ BL21(DE3) |
|  | BL21(DE3) GOLD pET24a-*fdpA* |  |
| ***C. difficile*** |  |  |
| 630∆*erm* |  | Laboratory stock |
| CDIP546 | 630∆*erm* *sigB*::*erm* | (Kint et al. 2017) |
| CDIP588 | 630∆*erm fdpA*::*erm* | pDIA6374 → 630∆*erm* |
| CDIP691 | 630Δ*erm* Δ*revRbr2* | pDIA6429 → 630∆*erm* |
| CDIP697 | 630Δ*erm* Δ*revRbr1* | pDIA6474 → 630∆*erm* |
| CDIP1369 | 630Δ*erm* Δ*fdpF* | pDIA6893 → 630∆*erm* |
| CDIP1595 | 630Δ*erm* Δ*fdpA* | pDIA6955 → 630∆*erm* |
| CDIP714 | 630Δ*erm* Δ*revRbr1* Δ*revRbr2* | pDIA6429 → CDIP697 |
| CDIP1370 | 630Δ*erm* Δ*fdpF-fdpA*::*erm* | pDIA6893 → CDIP588 |
| CDIP824 | 630Δ*erm* Δ*revRbr1* Δ*revRbr2 fdpA*::*erm* | pDIA6374 → CDIP714 |
| CDIP1401 | 630Δ*erm* Δ*revRbr1* Δ*revRbr2* Δ*fdpF* | pDIA6893 → CDIP714 |
| CDIP1402 | 630Δ*erm* Δ*revRbr1* Δ*revRbr2* Δ*fdpF fdpA*::*erm* | pDIA6893→ CDIP824 |
| CDIP659 | 630∆*erm* pFT47- P*_revRbr2_*-*SNAP^Cd^* | pDIA6459 → 630∆*erm* |
| CDIP670 | 630∆*erm* *sigB*::*erm* pFT47-P*_revRbr2_*-*SNAP^Cd^* | pDIA6459 → CDIP546 |
| CDIP658 | 630∆*erm* pFT47- P*_fdpF_*-*SNAP^Cd^* | pDIA6458 → 630∆*erm* |
| CDIP669 | 630∆*erm* *sigB*::*erm* pFT47-P*_fdpF_*-*SNAP^Cd^* | pDIA6458 → CDIP546 |
| CDIP1045 | 630Δ*erm* pFT47-Pσ^B^*_fdpA_*-*SNAP^Cd^* | pDIA6669 → 630∆*erm* |
| CDIP1047 | 630Δ*erm sigB*::*erm* pFT47-Pσ^B^*_fdpA_-SNAP^Cd^* | pDIA6669 → CDIP546 |
| CDIP767 | 630Δ*erm* pFT47-Pσ^A^/σ^B^*_fdpA_-SNAP^Cd^* | pDIA6517 → 630∆*erm* |
| CDIP774 | 630Δ*erm* *sigB*::*erm* pFT47-Pσ^A^/σ^B^*_fdpA_-SNAP^Cd^* | pDIA6517 → CDIP546 |
| CDIP1375 | 630∆*erm fdpA*::*erm* + pMTL84121*-*Pσ^A^/σ^B^-*fdpA* | pDIA6870 → CDIP588 |
| CDIP1181 | 630∆*erm fdpA*::*erm* + pDIA6103-*fdpA* | pDIA6806 → CDIP588 |
| CDIP800 | 630∆*erm* Δ*revRbr1* Δ*revRbr2*+ pMTL84121 Pσ^B^-*revRbr2* | pDIA6537 → CDIP714 |
| CDIP802 | 630∆*erm* Δ*revRbr1* Δ*revRbr2* + pMTL84121 Pσ^B^-*revRbr1* | pDIA6538 → CDIP714 |
| CDIP1449 | 630Δ*erm* Δ*fdpF fdpA*::*erm* + pMTL84121 Pσ^B^-*fdpF* | pDIA6388 → CDIP1370 |
| CDIP1522 | 630∆*erm* *fdpA*::*erm* complementation at the *fdpA* locus | pDIA6956 → CDIP588 |
| CDIP1450 | 630Δ*erm* Δ*revRbr1* Δ*revRbr2* Δ*fdpF* + pMTL84121 Pσ^B^-*fdpF* | pDIA6388 → CDIP1401 |
| CDIP1451 | 630Δ*erm* Δ*revRbr1* Δ*revRbr2* Δ*fdpF fdpA*::*erm* + pMTL84121 Pσ^B^-*fdpF* | pDIA6388 → CDIP1402 |
| Plasmids |  | Origins |
| pFT47 | SNAP*^Cd^*, Cm^R^-Tm^R^ | (Pereira et al. 2013) |
| pDIA6103 | pRPF185Δ*gusA* | (Soutourina et al. 2013) |
| pMTL007 | Clostron vector, Cm^R^-Tm^R^ | (Heap et al. 2007) |
| pMTLSC7315 | ACE vector, Cm^R^-Tm^R^ | (Cartman et al. 2012) |
| pMTL84121 | Cm^R^-Tm^R^ | (Heap et al. 2009) |
| pET20 | Amp^R^ | Novagen |
| pET24a | Km^R^ | Novagen |
| pDIA6388 | pMTL84121- Pσ^B^-*fdpF* | This work |
| pDIA6537 | pMTL84121- Pσ^B^-*revRbr2* | This work |
| pDIA6538 | pMTL84121- Pσ^B^-*revRbr1* | This work |
| pDIA6870 | pMTL8121-Pσ^A^/σ^B^-*fdpA* | This work |
| pDIA6806 | pDIA6103-*fdpA* | This work |
| pDIA6374 | pMTL007-CE5 *fdpA*-225s | This work |
| pDIA6893 | pMTLSC7315-∆*fdpF* | This work |
| pDIA6429 | pMTLSC7315-∆*revRbr2* | This work |
| pDIA6474 | pMTLSC7315-∆*revRbr1* | This work |
| pDIA6955 | pMTLSC7315-∆*fdpA* | This work |
| pDIA6459 | pFT47-P*_revRbr2_*-*SNAP^Cd^* | This work |
| pDIA6517 | pFT47- Pσ^A^/σ^B^*_fdpA_-SNAP^Cd^* | This work |
| pDIA6669 | pFT47-Pσ^B^*_fdpA_*-*SNAP^Cd^* | This work |
| pDIA6458 | pFT47-Pσ^B^*_fdpF_*-*SNAP^Cd^* | This work |
| pDIA6956 | pMTLSC7315- *fdpA* complementation at the same locus | This work |
| pDIA6635 | pET20-*revRbr1* | This work |
| pDIA6671 | pET20-*revRbr2* | This work |

Cartman, S. T., M. L. Kelly, D. Heeg, J. T. Heap, and N. P. Minton. 2012. Precise manipulation of the Clostridium difficile chromosome reveals a lack of association between the tcdC genotype and toxin production. Appl Environ Microbiol **78**:4683-4690.

Heap, J. T., O. J. Pennington, S. T. Cartman, G. P. Carter, and N. P. Minton. 2007. The ClosTron: a universal gene knock-out system for the genus Clostridium. J Microbiol Methods **70**:452-464.

Heap, J. T., O. J. Pennington, S. T. Cartman, and N. P. Minton. 2009. A modular system for Clostridium shuttle plasmids. J Microbiol Methods **78**:79-85.

Kint, N., C. Janoir, M. Monot, S. Hoys, O. Soutourina, B. Dupuy, and I. Martin-Verstraete. 2017. The alternative sigma factor sigmaB plays a crucial role in adaptive strategies of Clostridium difficile during gut infection. Environ Microbiol **19**:1933-1958.

Pereira, F. C., L. Saujet, A. R. Tome, M. Serrano, M. Monot, E. Couture-Tosi, I. Martin-Verstraete, B. Dupuy, and A. O. Henriques. 2013. The spore differentiation pathway in the enteric pathogen Clostridium difficile. PLoS Genet **9**:e1003782.

Soutourina, O. A., M. Monot, P. Boudry, L. Saujet, C. Pichon, O. Sismeiro, E. Semenova, K. Severinov, C. Le Bouguenec, J. Y. Coppee, B. Dupuy, and I. Martin-Verstraete. 2013. Genome-wide identification of regulatory RNAs in the human pathogen Clostridium difficile. PLoS Genet **9**:e1003493.

**Table S1B: Oligonucleotides used in this study**

| **Name** | **Sequence** |  |
| --- | --- | --- |
| IMV837 | AAAAAAGCTTATAATTATCCTTAAAAATCGACTATGTGCGCCCAGATAGGGTG | *fdpA*-225|226s-IBS primer |
| IMV838 | CAGATTGTACAAATGTGGTGATAACAGATAAGTCGACTATGTTAACTTACCTTTCTTTGT | *fdpA*-225|226s-EBS1d primer |
| IMV839 | TGAACGCAAGTTTCTAATTTCGGTTATTTTCCGATAGAGGAAAGTGTCT | *fdpA*-225|226s-EBS2 primer |
| IMV844 | CTTTACTGGAGTAGTAGATGAA | Verification intron *fdpA* |
| IMV845 | GACCATCTAAGTATAGTTTAGCA | Verification intron *fdpA* |
| IMV917 | TCATGAGATTATCAAAAGGGATAGACTTCTAAATATTTCCC | ACE *revRbr1* 5’ PCR1 rev comp vector |
| NK70 | ATTTCTTCATAATTACTTTCCTCCTAAA | ACE *revRbr1* 3’ PCR1 |
| NK71 | TTTAGGAGGAAAGTAATTATGAAGAAAGAAGCTAGACATGGTAAAGCATTC | ACE *revRbr1* 5’ PCR2 rev comp NK70 |
| IMV918 | GTAGAAATACGGTGTTTTTTAGCACAACTAACACTATAATTC | ACE *revRbr1* 3’ PCR2 rev comp vector |
| NK64 | GGTGCAGCATCTCCTTCATGTATA | ACE *revRbr2* 3’ PCR1 |
| IMV919 | TCATGAGATTATCAAAAGGAAGGAGGGGGCTTTATTGAAA | ACE *revRbr2* 5’ PCR1 rev comp vector |
| NK65 | TATACATGAAGGAGATGCTGCACCCTTAGGATTATTAAACAGACATTTTGG | ACE *revRbr2* 5’ PCR2 rev comp NK64 |
| IMV920 | GTAGAAATACGGTGTTTTTTGTTCATGGCAACAGTAACATC | ACE *revRbr2* 3’ PCR2 rev comp vector |
| NK58 | GTTATTTAGTCTCTTTATACCCTGCTTG | ACE *fdpF* 3’ PCR1 |
| IMV914 | TCATGAGATTATCAAAAGGGGACAATTTATCTTGATTTACTAG | ACE *fdpF* 5’ PCR1 rev comp vector |
| NK59 | CAAGCAGGGTATAAAGAGACTAAATAACGATTTTGAGGATATTATGAAGGCTG | ACE *fdpF* 5’ PCR2 rev comp NK58 |
| IMV915 | GTAGAAATACGGTGTTTTTTCACTTGAGTAAAGTCAGGATT | ACE *fdpF* 3’ PCR2 rev comp vector |
| CF115 | GGAATTCGCTCATAGATTAAGTACCATATC | Complementation *revRbr1* 5’ EcoRI |
| CF116 | ATAAGAATGCGGCCGCTTATTTTCCAAAATATCTATTTAATAATC | Complementation *revRbr1* 3’ NotI |
| CF113 | TCCCCCCGGGCAGAGGAGTCTTCATTAGAAAG | Complementation *revRbr2* 5’ EcoRI |
| CF114 | CCGCTCGAGTTATTTTCCAAAATGTCTGTTTAA | Complementation *revRbr2* 3’ XhoI |
| IMV699 | CCGCTCGAGAAAAGGGAACTGTATTG | Complementation *fdpF* 5’XhoI |
| IMV700 | CGGGATCCTTAAACCTATGTATGTA | Complementation *fdpF* 3’BamHI |
| NK259 | GCCGCTGTATCCATATGACCAAAATTCCTCCAAACAAGCTC | Complementation fdpA into pMTL8121 rev comp NK261 |
| NK260 | GTTTTCCCAGTCACGACGTTGTGTGATTAATGGCTATTTAAC | Complementation fdpA into pMTL8121 rev comp NK262 |
| NK261 | GGTCATATGGATACAGCGGC | Inverse PCR pMTL84121 |
| NK262 | AACGTCGTGACTGGGAAAAC | Inverse PCR pMTL84121 |
| IMV978 | AAGGCCTTATATAATGATAAATTTTAGGAGG | Complementation fdpA into ptet 5’ StuI |
| IMV979 | CGGGATCCGAGTTGAACTTAAAATTAGATTAGA | Complementation fdpA into ptet 3’ BamHI |
| CF80 | TGGTCATGAGATTATCAAAAGGTCCCTTCTCTATTAGGAGTTACA | Complementation *fdpA* 5’ rev comp vector/ACE *fdpA* PCR1 |
| CF81 | AAGTAACATACCCCATTTTGAGG | ACE *fdpA* 3’ PCR1 |
| NK180 | CCTCAAAATGGGGTATGTTACTTGGTATAACTATGGCTGAGAC | ACE *fdpA* 5’ PCR2 rev comp CF81 |
| CF83 | ATCGTAGAAATACGGTGTTTTTTAAGAAAATTACAAAGAACATGACC | Complementation *fdpA* 3’ rev comp vector/ACE *fdpA* PCR2 |
| IMV976 | AGGAATTCATAAAACTATTGTTTTACATGA | 5’ Pσ^B^*_fdpA_*-*SNAP^Cd^* EcoRI |
| IMV950 | CCGCTCGAGAGTTGTAAGTTGTCCCAA | 3’ Pσ^B^*_fdpA-_SNAP^Cd^* XhoI |
| CF23 | GGAATTCCAGAGGAGT CTTCATTAGAAAG | 5’ P*_revRbr2_*-*SNAP*^Cd^ EcoRI |
| CF24 | CCGCTCGAGAATTTTTATTTTTAAGTTTAATA | 3’ P*_revRbr2_-SNAP*Cd XhoI |
| CF21 | GGAATTCGTTTAGACAAAGATTTAGATGTTAC | 5’ P*_fdpF_*-*SNAP*^Cd^ EcoRI |
| CF22 | CCGCTCGAGTAAAATAGTTATTTAGTCTCTT | 3’ P*_fdpF_*-*SNAP*^Cd^ XhoI |
| IMV970 | GGAATTCCATATGAAAAAATTTGTTTGTACAG | 5’ *revRbr2* pET20 NdeI |
| IMV971 | CCGCTCGAGTTATTATTTTCCAAAATGTCTGTTTAA | 3’ *revRbr2* pET20 XhoI **(stop)** |
| IMV968 | GGAATTCCATATGAAGAAATTTGTTTGTACAG | 5’ *revRbr1* pET20 NdeI |
| IMV969 | CCGCTCGAGTTATTATTTTCCAAAATATCTATTTAATAATC | 3’ *revRbr1* pET20 XhoI **(stop)** |
